# Postoperative infection following anterior cervical fusion surgery caused by Prevotella oris: a case report

**DOI:** 10.3389/fsurg.2025.1555039

**Published:** 2025-08-29

**Authors:** Chenglong Wang, Yang Lei

**Affiliations:** ^1^ Spinal Surgery Department, Mianyang Orthopaedic Hospital, Mianyang, Sichuan, China; ^2^ Department of Spinal & Bone Tumors (1), The Affiliated Traditional Chinese Medicine Hospital of Southwest Medical University, Luzhou, Sichuan, China

**Keywords:** anterior cervical spinal fusion, postoperative infection, prevotella oris, high-throughput nucleic acid sequencing, case report

## Abstract

Postoperative infections following spinal fusion procedures are commonly caused by pathogens such as *Staphylococcus aureus*, coagulase-negative *Staphylococcus*, *Streptococcus* species, and *Escherichia coli*. However, cases involving *Prevotella oris* as a causative agent are rarely documented in the literature. We report a case of postoperative infection after anterior cervical spinal fusion surgery. After empirical antimicrobial therapy failed, high-throughput nucleic acid sequencing detected *Prevotella oris* in the patient's cerebrospinal fluid—a rare and previously unreported finding. Upon switching to a more sensitive antibiotic regimen, the patient's symptoms improved significantly. This case highlights an unusual etiology and provides a comprehensive documentation of the treatment process, aiming to assist clinicians in the diagnosis and management of similar infections.

## Introduction

Postoperative infections are a common complication following spinal fusion surgery. Although the incidence remains relatively low, these infections often require prolonged antimicrobial therapy, leading to extended hospitalization and significantly increased medical, social, and economic burdens (1, 2). Consistent studies have demonstrated that Staphylococcus aureus is the predominant pathogen responsible for postoperative infections following spinal fusion procedures (3, 4). However, other Gram-positive and Gram-negative bacteria can also contribute to such infections (5, 6). Here, we present a rare case of postoperative infection after anterior cervical spinal fusion surgery caused by *Prevotella oris*. By sharing the diagnostic and therapeutic approach, we aim to provide clinicians with valuable insights into the management of similar cases.

## Case report

**Figure 1:**
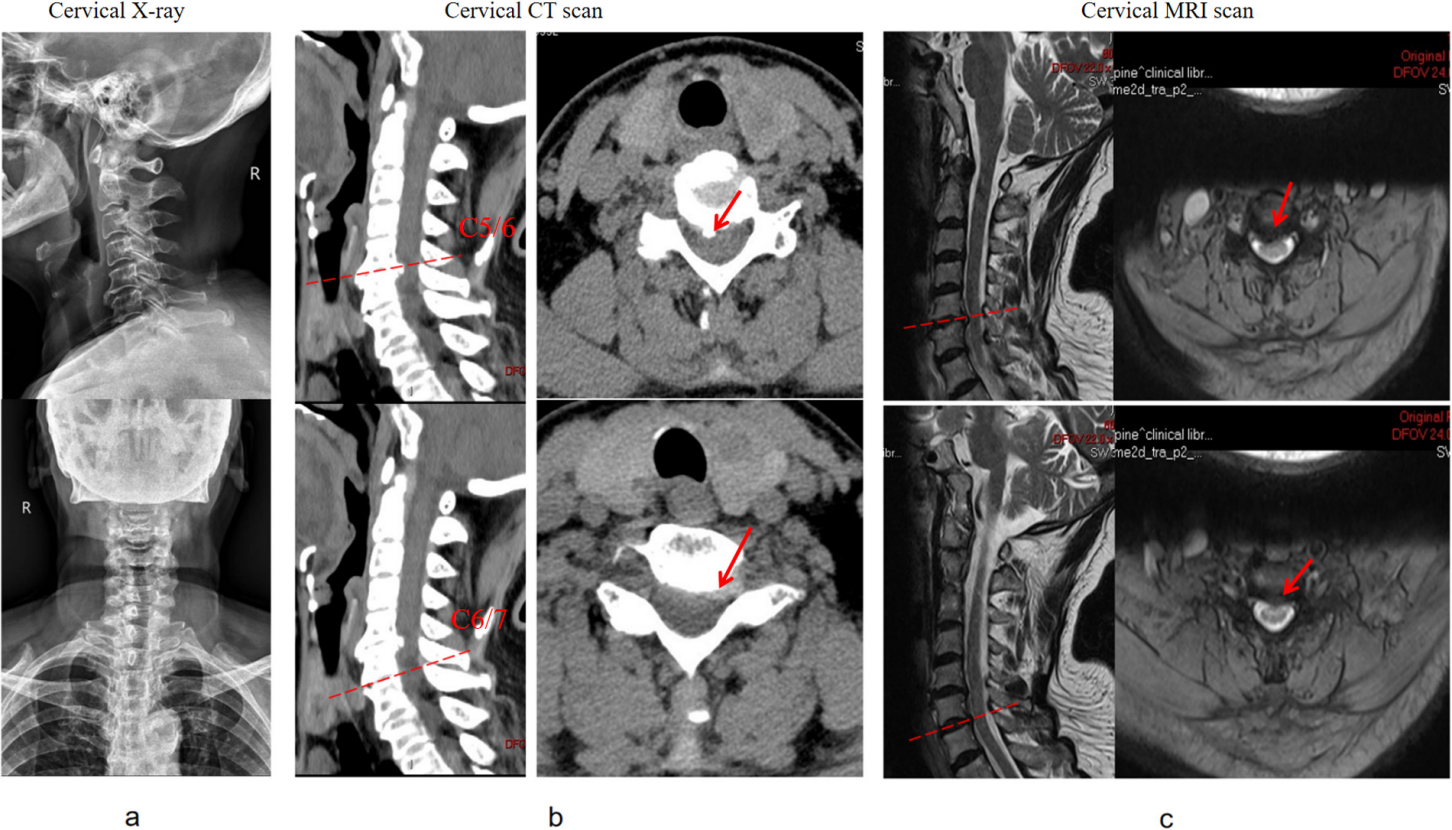
Preoperative imaging examination [including **(a)** X-ray, **(b)** CT scan, and **(c)** MRI] suggested herniation of the C5-7 intervertebral disc with compression of the dural sac and nerve root.

**Figure 2:**
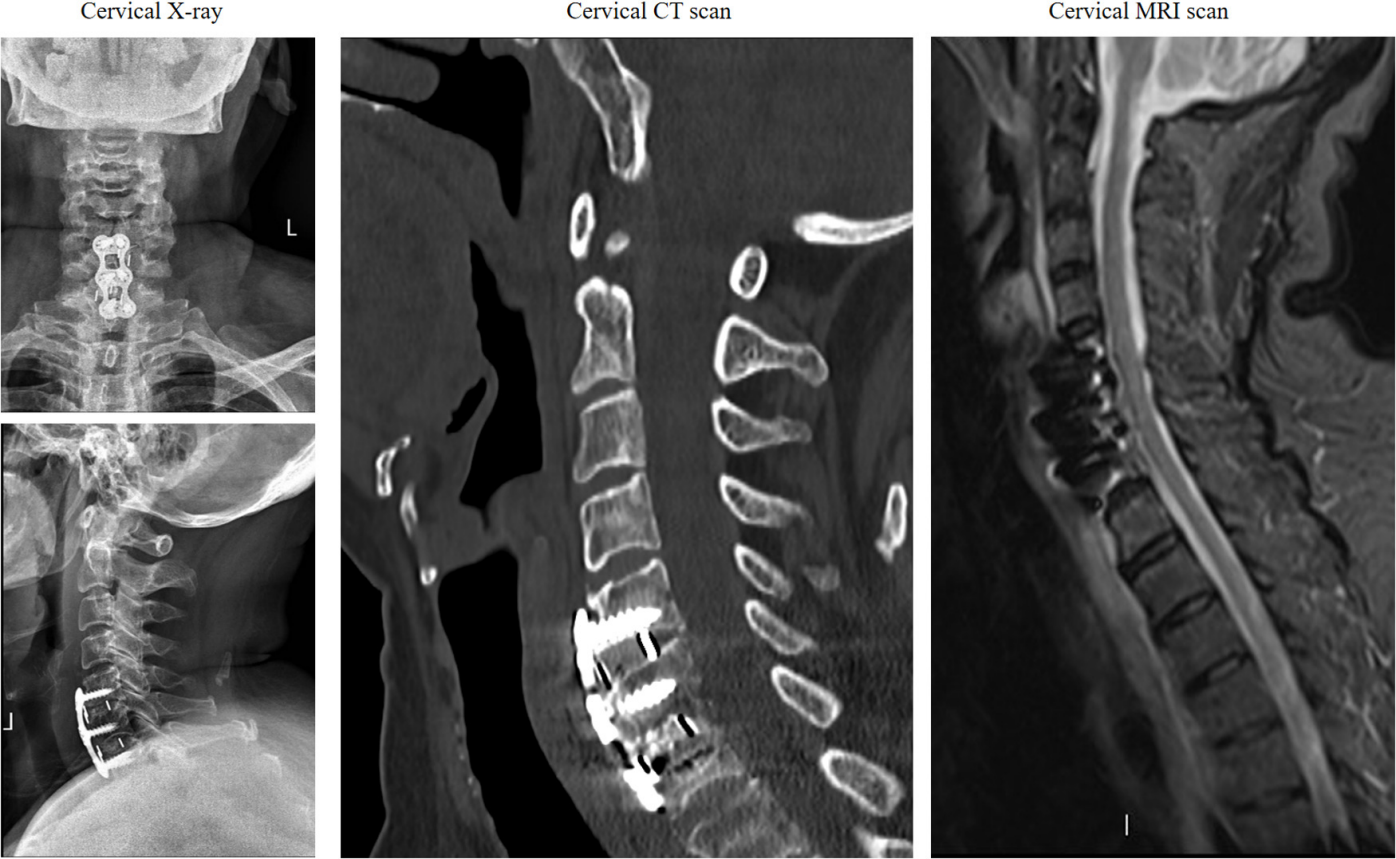
Postoperative cervical spine x-ray and CT imaging.

We present a case of postoperative infection in a 70-year-old female patient following anterior cervical spinal fusion. The patient was admitted for the management of neck pain accompanied by left upper extremity pain and numbness. Her medical history included hypertension and diabetes, both of which were managed with a regular medication regimen to control blood pressure and blood glucose levels. Physical examination revealed pronounced tenderness and muscle tension in the left cervical and shoulder regions, hypoesthesia in the lateral aspects of the left upper arm and forearm, and muscle strength graded 4 in the left triceps, wrist extensors, and finger extensors. Additionally, the bilateral foraminal compression tests and left brachial plexus traction test were positive. Preoperative imaging demonstrated a C5–7 intervertebral disc herniation (Figure 1), compressing the nerve roots and dural sac at the corresponding levels. Although the patient's symptoms were attributed to the left-sided herniation at C6/7, we prophylactically addressed the asymptomatic right-sided disc herniation at C5/6, considering both the increased risk of adjacent segment disease following fusion surgery and the potential limitations for future reoperations due to the patient's advancing age and progressively deteriorating physical condition. To alleviate her symptoms, a C5–7 anterior cervical discectomy and fusion (ACDF) was performed, which resulted in significant postoperative symptom relief. Postoperative imaging findings are shown in (Figures 2). Perioperative assessment confirmed the absence of surgically significant complications, including incidental durotomy, cerebrospinal fluid leakage, esophageal perforation, or laryngotracheal injury. This was corroborated by postoperative clinical indicators: absence of dysphagia, cervical swelling, or subcutaneous emphysema, coupled with minimal incisional drainage (<30 ml/day of serosanguineous fluid) and stable vital signs.

**Figure 3:**
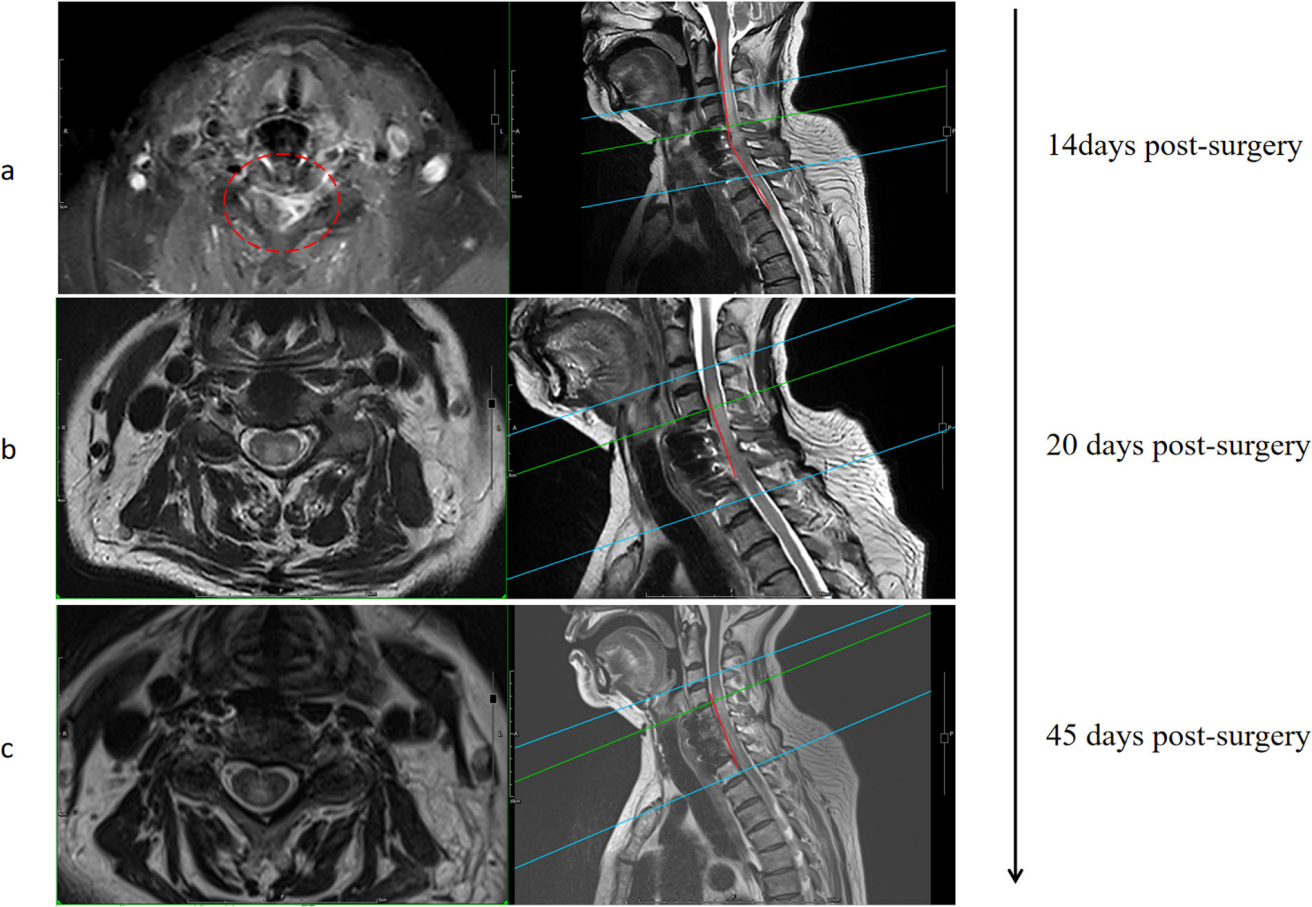
MRI imaging findings at different periods after the occurrence of axial pain after surgery **(a)** Imaging follow-up after the first empirical use of antibiotics **(b)** Imaging follow-up after antibiotic administration for one week after metagenomics revealed the causative organism **(c)** Imaging follow-up about 3 weeks after discharge.

Unfortunately, one week after surgery, the patient developed axial symptoms, including left neck and shoulder pain, stiffness, and muscle tension. Hematological results revealed a white blood cell count of 16.50 × 10^−9^/L, neutrophil count of 14.57 × 10^−9^/L (88.3%), procalcitonin level of 1.857 ng/ml, and C-reactive protein level of 120.0 mg/L, all suggesting infection. Since no pathogen was initially identified, empirical treatment with moxalactam sodium (0.5 g every 12 h) was initiated for one week. Despite this, the patient's condition worsened, with left upper limb weakness (about grade 3) and bilateral lower extremity weakness (about grade 3) developing. Follow-up hematological tests showed a white blood cell count of 17.00 × 10^−9^/L, neutrophil count of 15.69 × 10^−9^/L (92.3%), procalcitonin level of 1.229 ng/ml, and C-reactive protein level of 50.4 mg/L. MRI revealed spinal cord edema in the surgical area (Figure 3a). To determine the etiology, a lumbar puncture was performed for cerebrospinal fluid analysis, which showed a white blood cell count of 19.00 × 10^−6^/L. Pathogen nucleic acid high-throughput sequencing of the CSF identified Prevotella oris (relative abundance: 45.76%, confidence: 99%), a microorganism typically found in the oral and gastrointestinal microbiota.

After a multidisciplinary consultation involving neurology and pharmacy, the patient was treated with vancomycin hydrochloride (1.0 g every 12 h), piperacillin sodium/tazobactam (4.5 g every 8 h), and ornidazole (0.5 g every 12 h) for infection control. After 3 weeks of systemic antimicrobial therapy, hematological parameters normalized within institutional reference ranges (Table 1), indicating effective infection control and prompting discontinuation of further serological surveillance. At the last documented follow-up 6 weeks postoperatively, the patient exhibited resolution of left cervicobrachial pain, with improvement in muscle strength to grade 4 in the left upper limb and grade 4/5 in bilateral lower limbs. Concurrent MRI revealed regression of spinal cord edema (Figures 3b,c).

**Table 1:** Hematological results of patients at different time before and after surgery.

Inflammatory indicators	Preoperative	Postoperative day 1	Postoperative day 7	Postoperative day 15	Postoperative day 20
WBC (×10^−9^/L)	7.75	9.19	16.5	17.0	5.69
NEUT (%)	68.5	92.9	88.3	92.3	64.7
LYMPH (%)	23.7	6.3	8	3.6	23.6
NEUT (×10^−9^/L)	5.3	8.53	14.57	15.69	3.68
LYMPH (×10^−9^/L)	1.84	0.58	1.32	0.61	1.35
CRP (mg/L)	10.6	/	120.0	50.4	24.47
ESR (mm/h)	9.79	/	22.72	/	/
PCT (ng/ml)	/	/	1.857	1.229	/

## Discussion

Postoperative infections following spinal fusion are most commonly caused by Staphylococcus aureus. According to a report by the International Spine Research Society, the incidence of surgical site infections after spinal surgery is 1.2%, with Staphylococcus aureus (41.9%) and methicillin-resistant Staphylococcus aureus (MRSA) (17.0%) being the most prevalent pathogens (7). To our knowledge, no cases of Prevotella oris infection following spinal fusion surgery have been reported, based on a comprehensive search of the PubMed database. Only a few studies have identified Prevotella species as causative agents of brain abscesses, meningitis, or spinal canal infections (Table 2) (8–17). All findings were consistently identified through metagenomic and high-throughput sequencing, providing critical guidance for subsequent treatment decisions. Prevotella oris, a commensal bacterium primarily colonizing the oral cavity and gastrointestinal tract, was the detected pathogen. Upon hospital admission, the patient reported no complaints of oral lesions. The absence of dysphagia, cervical swelling, subcutaneous emphysema, coupled with minimal (<30 ml/day) serosanguineous (as opposed to clear) drainage from the surgical incision, effectively excluded esophageal perforation and cerebrospinal fluid leakage as potential postoperative complications in this case. Notably, prior studies have demonstrated a significant association between preoperative nasal bacterial colonization and postoperative surgical site infections in spinal procedures, suggesting potential dissemination of nasal flora to the wound (18). Given the close anatomical proximity between the cervical spine and the oral cavity, it is biologically plausible that Prevotella oris could disseminate from the oral cavity to the surgical site. Anterior cervical spine surgery (e.g., ACDF) requires dissection through the retropharyngeal space, where the posterior pharyngeal wall lies in close proximity to the prevertebral fascia. Intraoperative maneuvers (such as retraction or electrocautery) may cause microtrauma to the pharyngeal mucosa even without full-thickness perforation, potentially allowing oral flora to migrate through microscopic breaches into the surgical field. Furthermore, oropharyngeal procedures (including endotracheal intubation and suctioning) can induce transient bacteremia, enabling hematogenous bacterial seeding at the surgical site-particularly on implant surfaces.

**Table 2:** Characteristics of infections caused by Prevotella oris.

Diagnosis	Antibiotic	Time of therapy	Diagnosis method	Reference
Brain abscesses	Ceftriaxone and metronidazole	6 weeks	mNGS	Li et al. (8).
Meningitis, intraspinal infection	Metronidazole and meropenem	Metronidazole and meropenem for 2 weeks, and meropenem for 2 months	mNGS	Zhang et al. (9).
Multiple serous cavity effusions	Piperacillin-tazobactam and metronidazole	Several days	mNGS	Zhang et al. (10).
Bacteremia and liver abscess	Initially, piperacillin-tazobactam and levofloxacin; followed by piperacillin-tazobactam and metronidazole	10 days	16S rRNA gene sequencing	Cobo et al. (11).
Pleural infection	Metronidazole	5 days	pleural pus culture	Viswanath et al. (12).
Pneumonia associated with pleural empyema	Cefotaxime, vancomycin and clindamycin	5 days	16S rRNA gene sequencing	Fernández et al. (13).
Necrotizing pneumonia	Cefotaxime and vancomycin	18 days	16S rRNA gene sequencing	
Retropharyngeal abscess and severe pneumonia	Meropenem and moxifloxacin	Intravenous meropenem and moxifloxacin for 2 days, followed by oral moxifloxacin for 2 weeks	mNGS	Li et al. (14).
Pericarditis	Initially, ceftriaxone and doxycycline; subsequently, ampicillin/sulbactam	Not mentioned	16s rRNA	Carmack et al. (15).
Bacteremia and sepsis	Ampicillin and metronidazole	Not mentioned	Blood culture	Bein et al. (16).
Cervical epidural abscess and meningitis	Penicillin, ceftriaxone and metronidazole	Intravenous fosfomycin, ceftriaxone and metronidazole for 3 weeks, followed by oral metronidazole for 8 weeks	Blood culture	Frat et al. (17).

We aimed to summarize and share the insights from this case to guide clinical diagnosis and treatment. First, when infection is suspected after spinal fusion surgery, clinical manifestations such as deterioration or fever (though absent in this case) may occur. Routine diagnostic tests should include a complete blood count, erythrocyte sedimentation rate (ESR), C-reactive protein (CRP), procalcitonin (PCT), cervical x-rays, CT scans, and cervical MRI (including contrast-enhanced MRI). Second, high-throughput genomic sequencing has increasingly identified rare pathogens in recent years, aiding clinicians in accurate pathogen identification and guiding standardized treatment strategies. Although high-throughput genomic sequencing holds promise for rapid diagnosis, its findings must be interpreted in concordance with clinical presentation, laboratory results, and imaging studies. Finally, early initiation of appropriately targeted antibiotics significantly improves patient prognosis.

## Data Availability

The raw data supporting the conclusions of this article will be made available by the authors, without undue reservation.
